# Single step fabrication of Silicon resistors on SOI substrate used as Thermistors

**DOI:** 10.1038/s41598-019-38753-x

**Published:** 2019-02-26

**Authors:** Serena Rollo, Dipti Rani, Wouter Olthuis, César Pascual García

**Affiliations:** 1 Nano-Enabled Medicine and Cosmetics group, Materials Research and Technology Department, Luxembourg Institute of Science and Technology (LIST), Belvaux, Luxembourg; 2 BIOS Lab on Chip Group, MESA+Institute for Nanotechnology, University of Twente, Enschede, The Netherlands

## Abstract

Temperature sensing is one of the important features of Micro Electro Mechanical Systems and a monolithic integration provides advantages for both fabrication simplicity and performance. The use of Silicon On Insulator substrates allows simple fabrication of integrated wires that can be used as thermistors. We fabricated rectangular and triangular silicon wires with different dimensions in a single step fabrication process based on the wet etching of a <110> Silicon On Insulator substrate. We determined the experimental resistivity of the two kinds of devices and tested their performance as thermistors in a temperature range between 24 and 100 °C. The accuracy and normalized sensitivities of our devices were 0.4 °C and 0.3–0.5%/°C, respectively. The potential of the proposed method resides in the possibility of having devices with different shapes in a single straightforward process.

## Introduction

Online and real-time measurements of temperature are fundamental for many applications in different domains ranging from bio-sensing, environment, electronics, etc.^1,2^. In this context the drive for miniaturisation demands possibilities to integrate thermometers together with other electronic components. The most often used options include thermocouples^3,4^ and resistive thermometers (thermistors)^5,6^, the later ones offering robust and cost-effective manufacturing with a considerably good stability and accuracy (reaching <0.01 °C for metallic thermistors). Silicon thermistors, although they are not as sensitive as the metallic, can be attractive because they can be integrated into electronic circuits like Complementary Metal Oxide Semiconductor (CMOS) circuits, MEMS, and Lab-on-a-Chip (LoC) devices taking advantages of the well-established silicon fabrication techniques. Furthermore, the monolithic integration of electronics and sensors with an optimised design that improves the surface area in contact with the object to measure is a way to improve the thermalization and the performance of the thermometers.

Since the implementation of fin field effect transistors (FinFETs) for high speed electronics^7,8^ and the development of new MEMS^9–11^, Silicon on Insulator (SOI) substrates based technologies are replacing conventional silicon substrates ones for the most demanding applications. In the last three years the global SOI market has doubled due to the advantages related to the buried oxide (commonly *SiO*_2_) underneath the top silicon device layer, which reduces parasitic capacitances and leakage currents due to the different electrical and thermal conductivities of *Si* and *SiO*_2_ (twelve and two orders of magnitude respectively larger in silicon). The difference in the thermal conductivity also opens the door to new architectures in which the temperature sensor can thermalize with other device components (like liquids in microfluidic devices, or a thermally conductive coating) or the own operating circuit, and not mainly with the substrate as traditional thermometers.

Tailoring the shape of a thermometer with anisotropic etching can be appealing in order to have the flexibility to optimize the surface area in contact with the object to measure. Anisotropic etching can be accomplished by either dry or wet processes^12,13^, both requiring a mask with high selectivity relative to the substrate. Dry processes are used for anisotropic etching of high aspect-ratio structures. However, they are known to induce unwanted defects and roughness coming from the ion bombardment. On the other hand, wet etching of *Si* can provide both anisotropic and isotropic structures, even in the same process, due to the dependency of the etching rates on the crystallographic orientation of the planes exposed to the solution^14–16^. Wet etching also provides smoother surfaces and less defects. Producing different structures within a single process can also increase the efficiency and reliability of the fabrication, reducing variability between devices. <100> oriented substrates are commonly used for the fabrication of triangular structures by wet etching^17,18^. Fabrication of rectangular structures can be achieved on the same substrates by dry etching^19^. Structures with other shapes can also be obtained by subsequent steps of oxidation and etching of the grown oxide^18^. However wet etching of <110> avoids some of the damaging introduced by dry etching, and can create simultaneously different shapes within the same process.

In this paper we present a single step fabrication process of silicon resistors with different shapes on a SOI chip, following the study of the etching rates of tetramethylammonium hydroxide (TMAH) solutions on different crystallographic directions on the <110> silicon device layer, to produce both triangular and rectangular devices. We tested them as silicon thermistors and extrapolated the first order thermal coefficient. Additionally we calculated the accuracy of our devices (~0.4 °C) and the normalized sensitivity (~0.3–0.5%/°C), which are comparatively better than other silicon thermistors^20,21^. The proposed method offers customization possibilities to produce devices with different geometries and contact areas to monitor better the temperature dependent process of interest.

## Materials and Methods

We used a 3 inches prime quality p-doped SOI substrate from Ultrasil Corporation, with a 2 ± 0.5 μm thick silicon device layer <110> oriented, and a 1 μm thick buried *SiO*_2_, both for the optimisation of the etching process producing different shapes, and for the final fabrication of the silicon thermistors.

### Sample patterning

The as bought substrate was sent for dicing into 1cmx1cm chips to Siegert Wafer. After dicing we cleaned the chips with solvents (acetone, isopropanol and deionized water). Then a 50 nm film of *SiO*_2_ was grown as masking material by thermal oxidation of the silicon top layer at 1000 °C and atmospheric pressure, with a 200 sccm flow of oxygen during 190 seconds. We used a FEI Helios electron microscope with the electron beam energy of 30 keV to expose specific designs on a negative resist ma-N2043 spun at 2000 rpm. The samples were developed for 1 minute in ma-D developer with manual stirring. The resulting resist patterns were then transferred into the underlying *SiO*_2_ by Reactive Ion Etching (RIE) using CF_4_ plasma (19 sccm) with a power of 25 W and a pressure of 75mTorr.

To eliminate the irregularities produced during RIE we carried out the etching until few nm of *SiO*_2_ were left outside the patterns. Thereafter we removed the resist mask by keeping the sample into remover PG for 10 minutes and rinsing it well with isopropanol and deionized water. After that we removed the leftover *SiO*_2_ using *HF* (2%) treatment for 1 minute which left a flat clean surface of *Si*. Before the *HF* treatment of the leftover *SiO*_2_ we removed the resist mask keeping the sample in remover PG for 10 minutes and rinsing it with isopropanol and deionized water.

### TMAH wet etching

A wt.25% TMAH solution, with addition of vol.8.5% of isopropanol (IPA) was used as etchant. We heated the solution to 43 ± 1 °C and performed the etching under stirring at 250 rpm. The process was carried out in a borosilicate beaker with 35 ml of etching solution placed on top of the stirrer-hot plate. The temperature was controlled by an external thermometer immersed in the etching solution.

### Calibration of the <110> silicon wet etching rates

In order to have information about the etched profiles and rates on the SOI <110> we made a pattern consisting of a set of crosses in which the arms of the crosses rotated by 0.5° closer in each step. The dimensions of the crosses arms were 0.8 × 18 µm^2^. In each cross we obtained the information from four angles with respect to the primary flat (see Supporting Information). For the calibration of the etching rates in the selected orientations resulting in rectangular and triangular walls we prepared samples with patterned rectangles 40 μm long and widths ranging from 500 nm to 3 μm, in steps of 500 nm, which were etched at different etching times with intervals of 2 minutes up to the complete etching of the device layer. The obtained information was finally used to prepare the sample with the rectangular and triangular resistors to be applied as temperature sensors.

### Ohmic contacts

In order to measure the resistors by an external measurement set-up we needed to fabricate ohmic contacts. These were patterned by a Mask-less Laser Aligner (MLA150) set-up (Heidelberg Instrument) with a wavelength of 365 nm exposing on a double layer resist consisting of LOR10A and Shipley1813 spun at 1000 and 2000 rpm for 30 and 60 seconds respectively, to obtain a thickness of ≈4 μm for the complete coverage of the 2 μm high patterned silicon structures. Then a stack of *Ti(5* *nm)*/*Al(160* *nm)*/*Au(50* *nm)* was deposited by electron beam evaporation under high vacuum (<10^−7^ mTorr). The annealing was carried out under vacuum at 400 °C for 5 minutes in a reductive atmosphere made by *N*_2_/*H*_2_ (5%). Then, wider metallic leads for electrical characterization were defined by a second MLA process. For these, we first evaporated a layer of 5 nm of *Ti* and 50 nm of *Au* by electron beam evaporation, followed by 50 nm of *Au* deposited by conformal sputtering, to assure electrical continuity between the leads and the ohmic contacts.

### Electrical and Thermal characterization

Electrical transport experiments were carried out using a Keithley 2614HB DC source-meter. Current-Voltage (IV) curves were acquired for multiple devices with different shapes and dimensions using two wire characterisation. The thermal characterization of the sample was made on the CascadeMicrotech PM8 probe station with a heated sample holder. The temperature was set to the desired value from the controller of the heating stage. We monitored the temperature of the substrate using a type K thermocouple from DOSTMANN electronic GmbH fixed on a silicon chip close to the sample using Kapton® tape. The IV curves were recorded only after the stabilisation of the temperature as measured from the probe.

## Results and Discussion

### Design and fabrication wires with a single process

**Figure 1:**
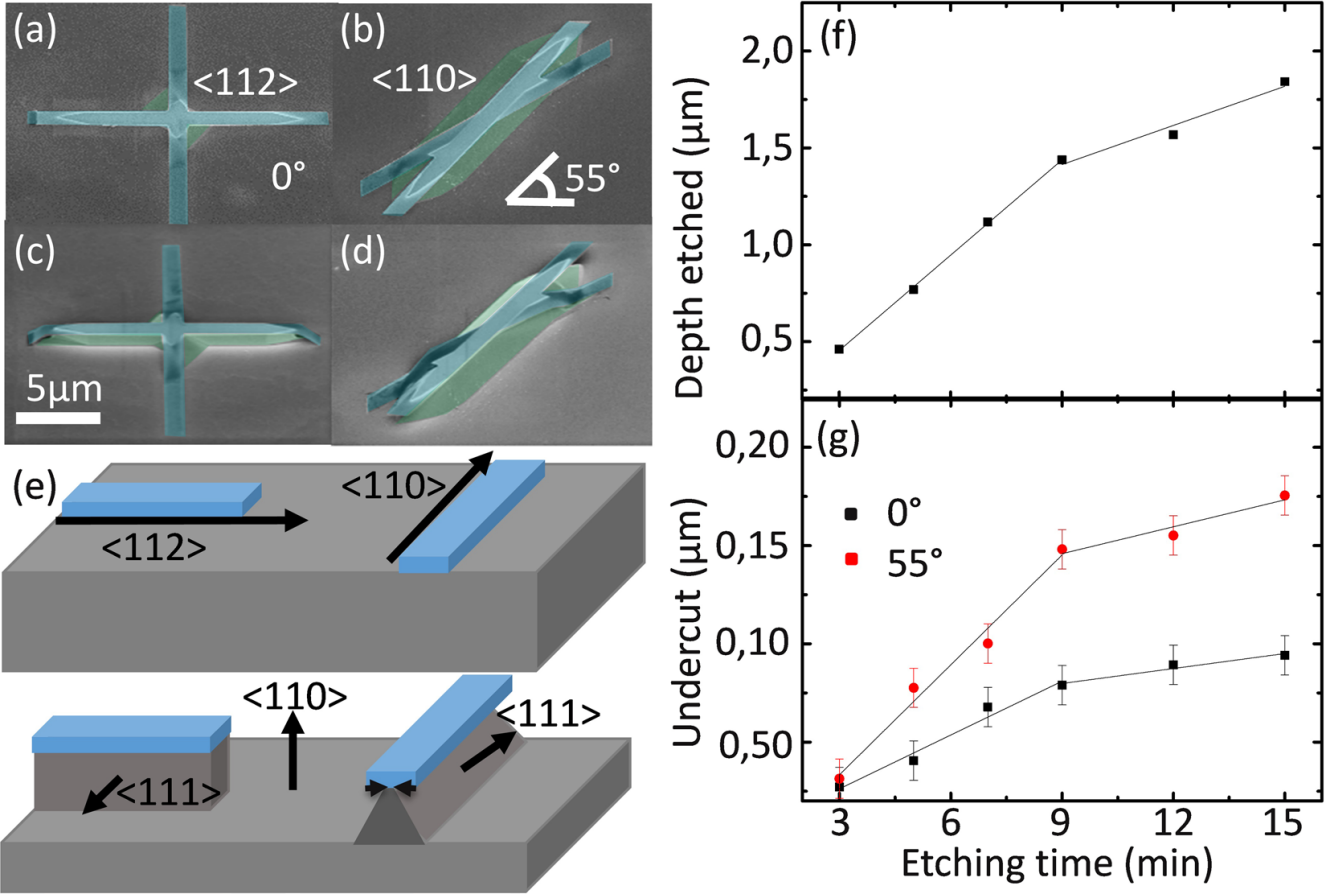
Top view (**a,b**) and tilted (**c,d**) scanning electron microscope pictures of the crosses with arms aligned in the two directions giving rectangular and triangular etched profiles. The SiO_2_ mask is shadowed in blue. (**e**) Schematic of etching masks aligned in the chosen crystallographic directions and resulting etching profiles. (**f**) Depth etched in the direction perpendicular to the substrate and (**g**) undercut observed for the directions parallel to the primary flat and 55° that resulted in rectangular and triangular wires, respectively.

The anisotropic etching of *Si* is well understood^22–25^ but a recipe to obtain different shapes in a single step process requires the calibration of the simultaneous etching rates depending on the plane orientation. We analysed the different profiles obtained by different pattern orientations from the etched crosses described in Materials and Methods. Figure 1(a) to (d) show representative scanning electron microscope (SEM) pictures of crosses ((a) and (b) are the top view and (c) and (d) are tilted images). The initial *SiO*_2_ mask is shadowed in blue, while the etched profile is shadowed in green. The directions parallel (<112>) and at 55 ± 2° (<110>) with respect to the primary flat resulted in perpendicular and 36° tilted walls (<111>) with respect to the substrate, respectively. They also showed smoother etched surfaces compared to the other orientations. These results are consistent with the ones reported in literature^26,27^. In Fig. 1(e) we schematically represent the high symmetry etching planes and their resulting etching profiles depending on the alignment of the mask on the top silicon device layer. As expected from TMAH wet etching studies of *Si*, the plane along the <110> direction was etched faster, while the planes along the <111> direction were almost unaffected^23^.

Using samples specifically designed with the two selected orientations (section 2.3) we measured the etching rates and undercut with a KLA Tencor P-17 profilometer and SEM, respectively. Figure 1(f,g) show the results of six samples etched at different times with intervals of 2 minutes. Initially the vertical etching rate was 163.9 nm/min, and the undercut 9.1 nm/min and 18.1 nm/min for the <112> and <110> orientations respectively. After nine minutes these values decreased to 67.3 nm/min, 2.5 nm/min and 4.6 nm/min, respectively. We attribute the slowdown of the rates to the saturation of the etching solution, as in other experiments we recovered the initial rate by refreshing the etchant. As the changes in the etching rates were proportional in all directions the final shapes and roughness were unaffected. Therefore, we used the same solution throughout the whole etching process for the fabrication of the *Si* resistors.

**Figure 2:**
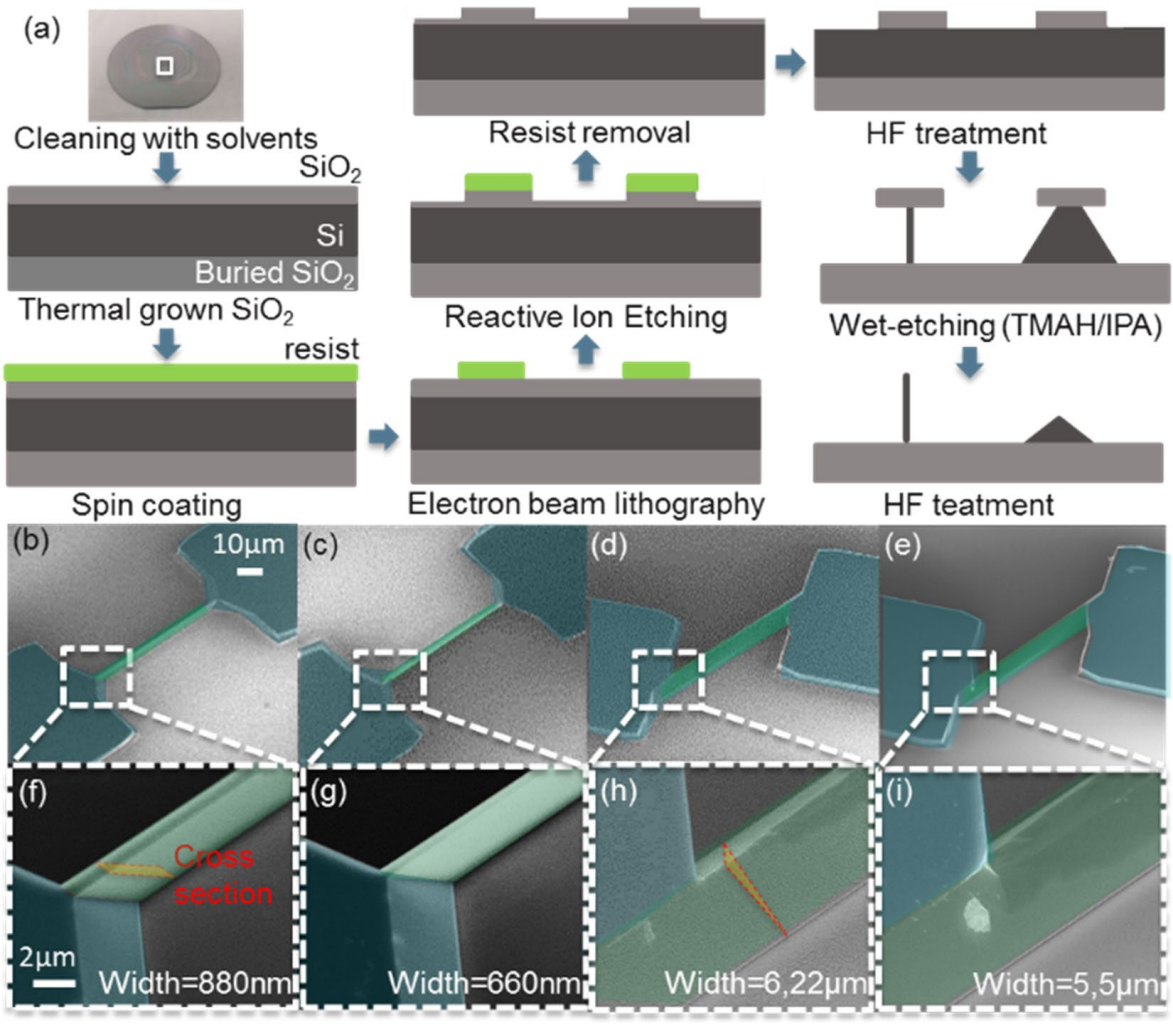
(**a**) Schematic representation of the fabrication protocol for the realization of rectangular and triangular wires. (**b**) to (**e**) Top view SEM pictures of representative rectangular and triangular wires (**b–d,e**), respectively). (**f**) to (**i**) magnifications showing the detailed shape of the wires in (**b**) to (**e**). The wires and the contacts have been shadowed in the SEM pictures in green and blue respectively. The cross section in perspective has been indicated in figures (**f,h**). The scale bars are common in both rows.

*Si* resistors with different shapes were obtained in a single step process combining different orientations of the mask and following the protocol schematized in Fig. 2(a). Briefly, we cleaned the sample with solvents (acetone, isopropanol and deionized water) before the growth of the *SiO*_2_ layer used as mask. We spin coated ma-N negative resist and patterned the wires with electron beam lithography. The patterned designs were transferred into the *SiO*_2_ by RIE leaving a thin layer of *SiO*_2_ outside the pattern areas. We removed the leftover resist with solvents. After that, we performed a 1 minute HF treatment to remove the remaining *SiO*_2_ outside the pattern areas and open a flat surface on the *Si* to be etched. Then we proceeded with TMAH wet etching. At the end, we performed a *HF* treatment to fully remove the leftover *SiO*_2_.

To simplify the electrical characterisation we chose the dimensions of the wire that would produce a resistance orders of magnitude bigger than the typical one from contacts (tenths of KΩ versus few hundreds of Ω). We fixed the length of the wire to 60 μm and we added square contact pads of 60 × 60 μm^2^. Based on resistivity provided by the fabricant (0.12 Ω·cm) we estimated that we needed widths after etching in the range of few hundreds nm and a few µm for the rectangular and triangular structures respectively to obtain the desired resistance. To achieve these dimensions we needed 23 minutes of etching time to ensure a complete etching of the top silicon layer in the vertical direction resulting in undercuts of 240 nm and 460 nm for the <112> (rectangular wires) and <110> (triangular wires) orientations, respectively. We lithographed masks with widths from 800 nm to 1.1 μm, and 200 nm to 400 nm in steps of 100 nm, aligned in the two chosen directions and connected to two squared contact pads. Small approaching pads connecting the silicon wires and the pads were designed with a triangular footprint with angles of ≈54.7° and ≈35.3° with respect to the primary flat to maintain smooth slanted walls between the channel and the walls coming from the etching of the pads. At the end of the process we obtained silicon wires with widths between 660 and 880 nm, and 5.5 and 6.2 μm for the rectangular and triangular structures respectively.

Figure 2(b) to (i) are representative SEM images corresponding to the bigger and smaller devices for both the rectangular and the triangular wires. Figure 2 (b) to (e) show the top view of whole devices with the contact pads and wires shadowed in blue and green, respectively. Figure 2(f) to (i) show zoomed in regions close to the contacts and the cross section for the bigger wires of each class. It can be noticed that after the wet etching the rectangular wires have the same height as of the contacts (see in Fig. 2(f) and (g)), while that of the triangular wires decreased. This makes that for similar footprint rectangular and triangular wires would have very different cross sections, and consequently different total resistance.

### Transport and temperature performance of the silicon wires

**Figure 3:**
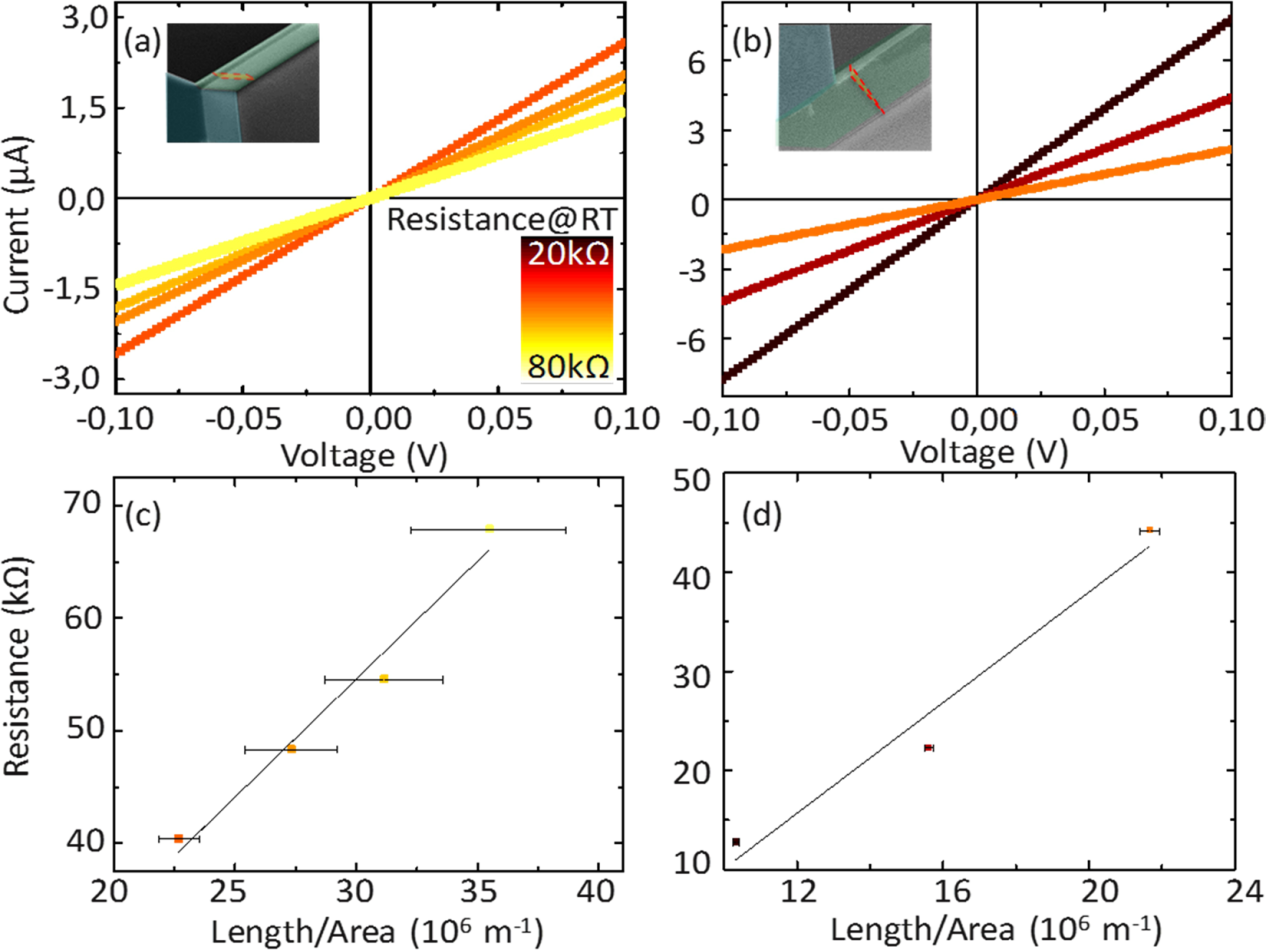
(**a,b**) Two probes current versus voltage (IV) curves of the rectangular and triangular wires, respectively. Each curve has a different colour according to the resistance scale in (**a**). (**c**,**d**) Plot of the resistances calculated from the curves in (**a**,**b**) versus the dimensional parameter length divided by area of the wires, for rectangular and triangular structures respectively. Error bars represent the propagation of the experimental uncertainty of the dimensions (length, area).

Figure 3(a,b) show the two probes current versus voltage (IV) curves of the rectangular and triangular wires, respectively. The IV curves are linear crossing the zero of both axis, reflecting a good ohmic behaviour. We have identified each with a colour associated to the room temperature (RT) resistance using the scale in Fig. 3(a), which is also linked to their dimensions. The different aspect ratio between the wires provided a large variety of cross sections consequently having different resistance, yet the smallest triangular wire could be used in comparison with the two rectangular wires with middle resistances. The total measured resistances were in the range of 39.7 to 66.9 and 12.8 to 44.2 kΩ for the rectangular and triangular wires, respectively. In Fig. 3(c,d) we plotted the value of these resistances versus a dimensional parameter length divided by area for rectangular and triangular structures respectively. The errors come mainly from the propagation of the experimental uncertainty of the dimensions on the resistance. We obtained higher error values for the rectangular wires with high aspect ratio due to their small widths. From the slope in Fig. 3(c,d) the experimental resistivity were 0.19 ± 0.04 Ω·cm and 0.25 ± 0.01 Ω·cm for the rectangular and triangular structures, respectively. These values are slightly higher than expected according to the dimensions of the wires and the fabricant resistivity. We attribute the small difference with respect to the value of the provider to the impact of the fabrication defects at the surface. We noticed that the resistivity of the triangular wires was higher, which is consistent with a higher roughness in the slanted etched planes observed in SEM images (see representative images in SI).

**Figure 4:**
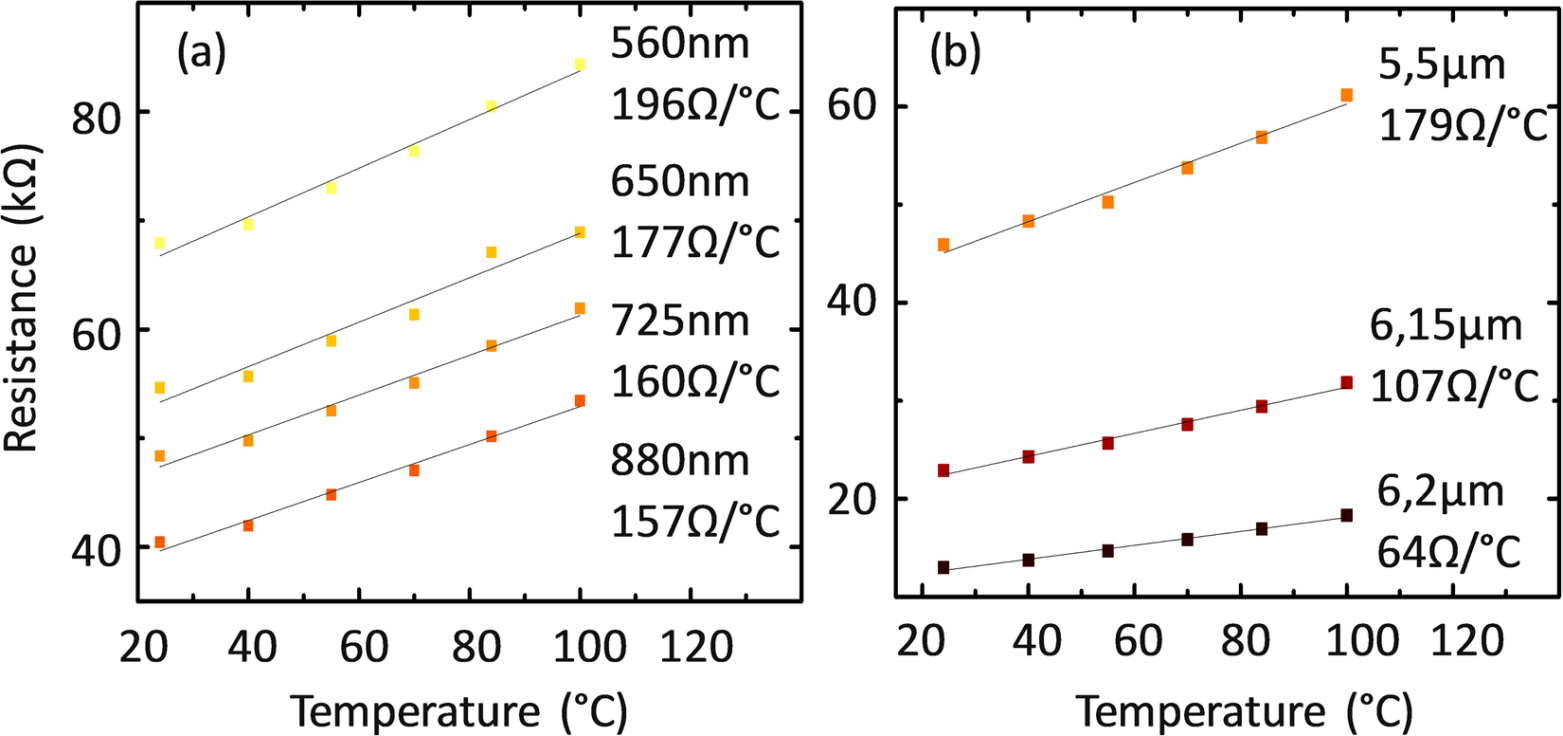
(**a,b**) Plot of the resistance versus temperature for the rectangular and the triangular wires respectively. The linear fitting was used to estimate the first order thermal coefficient of our devices. Each line corresponds to a specific device with the base width specified along with the first order thermal coefficient.

Temperature measuring is a possible application of any resistor. To study the performance of our wires we recorded the IV behaviour at different temperatures between 24 °C and 100 °C and extracted the resistance (see raw data in SI) reported in Fig. 4(a,b) for the square and triangular wires, respectively. The wires are identified with the colour associated to their RT resistance according to the colour map introduced in Fig. 3. We observed a linear behaviour in all the cases and therefore we used the slope to extract the first order temperature coefficient (*k*) with a linear fitting obtaining values between 64 and 196 Ω/°C as indicated in Fig. 4. The first order thermal coefficient (*k*) decreases with increasing dimensions of the wires, and is linked to the original resistance of the wires. The accuracy of the wires as thermometers was calculated based on the uncertainty in *k* from the fit in Fig. 4 and was found to be ±0,4 °C for both the rectangular and triangular wires. The main contribution to *k* is attributed to the variation of the resistivity and in particular to the change in the mean free path. We observed this as the change in resistivity was consistent with data in literature^28^ and as the thermal expansion coefficient of silicon is 2.6 × 10^−6^ °C^−1^, and its effect was estimated to contribute in 1 ppm to the variation of the resistance in the considered temperature range (see details in supporting information). We also estimated the normalized temperature sensitivity of our devices, which represents how much the resistance varies in percentage for one degree variation of temperature. This was accomplished by dividing the obtained first order thermal coefficient by the room temperature (RT) resistance. Results are shown in Table 1, and are comparable, even better regarding triangular thermistors, than what has been reported in literature^20^.

**Table 1:** Normalized temperature sensitivity for our rectangular and triangular cross section silicon thermistors.

Rectangular wires		Triangular wires	
*k (Ω/°C)*	*ΔR/R(%/°C)*	*k(Ω/°C)*	*ΔR/R (%/°C)*
157	0.39	64	0.5
160	0.3	107	0.48
177	0.37	179	0.4
196	0.29		

## Conclusions

We presented a single process for the simultaneous fabrication of rectangular and triangular silicon structures on a <110> SOI substrate and we tested their performance as silicon thermistors. The devices show a linear IV characteristics, with room temperature resistances in the range of a few kilo ohms. Their behaviour as resistors was well predicted from the bulk conductivity and the apparent dimensions of the devices. Results of measured resistivity showed that the process introduced some defects. In particular, triangular devices showed higher resistivity, which can be a result of higher roughness observed in the surface. Surface roughness could be further improved for a better performance of the device with the optimisation of the etching solution for example, by the use of surfactants^29^. The resistance of the devices varied linearly with temperature in the explored range (RT to 100 °C), with a first order thermal coefficient *k* between 63 and 196 Ω/°C, obtaining a change of the resistivity of 13.5% in the temperature range investigated. The higher resistances corresponded to higher values of *k* and for similar room temperature resistance the first order temperature coefficient did not depended on the shape of the wire. We estimated the accuracy of the fabricated silicon thermistors as 0,4 °C, and the normalized temperature sensitivity for each device which were found to be in accordance or even better than normally expected for silicon based temperature sensors^20,21^.

We presume that due to the different ratio of surface area exposed to the substrate or to the surrounding, these devices can be optimise to have better thermal coupling efficiency with the substrate, the electronic circuit or with the outside. High aspect ratio rectangular thermistors devices could be better applied for tracking the temperature of the surrounding medium while triangular cross section ones would better thermalize with the connected circuits or the substrate.

## Supplementary information

Supporting information
