# Supplementary material for: Single step fabrication of Silicon resistors on SOI substrate used as Thermistors
Source: Sci Rep. 2019 Feb 26;9:2835. doi: 10.1038/s41598-019-38753-x (PMC6391396; doi:10.1038/s41598-019-38753-x)
Supplement: Supplementary file 1 — Supporting information [file 41598_2019_38753_MOESM1_ESM.docx]

**Single step fabrication of Silicon resistors on SOI substrate used as Thermistors**

Serena Rolloᵃ^,^ᵇ, Dipti Raniᵃ, Wouter Olthuisᵇ, César Pascual Garcíaᵃ

ᵃ Nano-Enabled Medicine and Cosmetics group, Materials Research and Technology Department, Luxembourg Institute of Science and Technology (LIST), Belvaux, Luxembourg

ᵇBIOS Lab on Chip Group, MESA+ Institute for Nanotechnology, University of Twente, Enschede, The Netherland

**Supporting information**

1. **Study of the etching profile on 110 silicon on insulator**


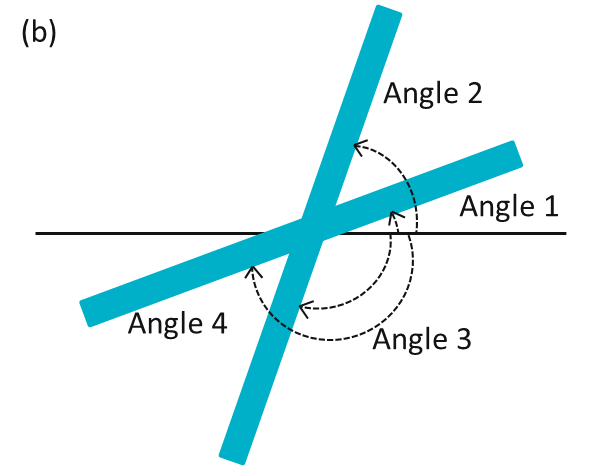
*
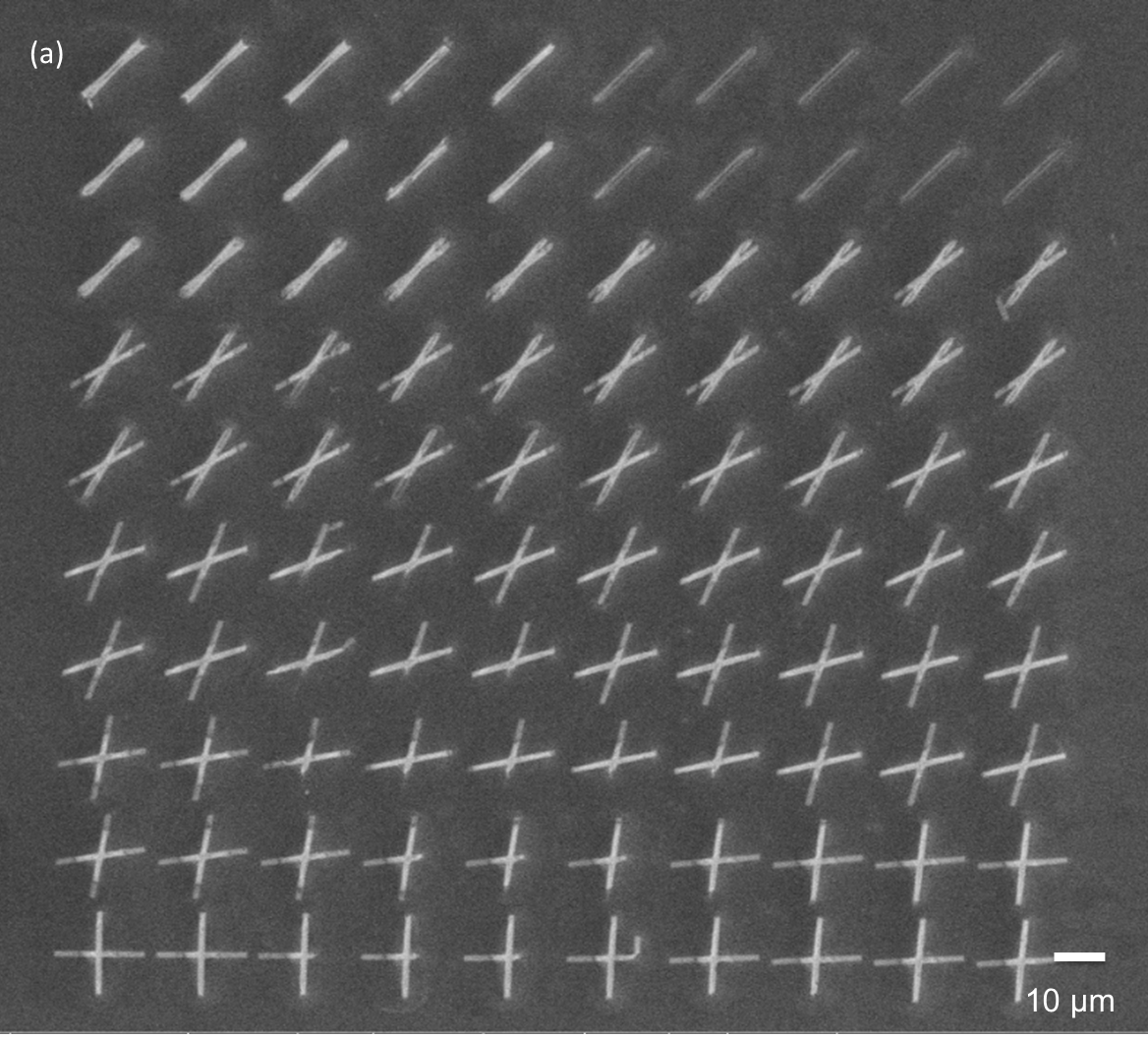
*In fig. 1 (a) the crosses which have been patterned on the substrate for the study of the etching profiles as explained in section 2.3 are shown. Figure 1 (b) is a schematic of a cross and the four angles about which it is possible to obtain information after etching. This applies for each cross.

**Figure 1.** (a) Pattern with crosses with arms oriented along all the 360° angles for the preliminary study of the etching profile on 110 SOI substrate. (b) Schematic representing a cross and the four angles about which it is possible to get information after etching.

Figure 2 (a) and (b) are high contrast SEM pictures of the etched walls with perpendicular and tilted angles respect to the substrate, respectively from a representative sample. Figure 2 (c) shows the two etched planes from one of the devices with vertical walls, at the junction. The pictures show the apparent higher roughness is observed for tilted walled as compared for perpendicular walls.


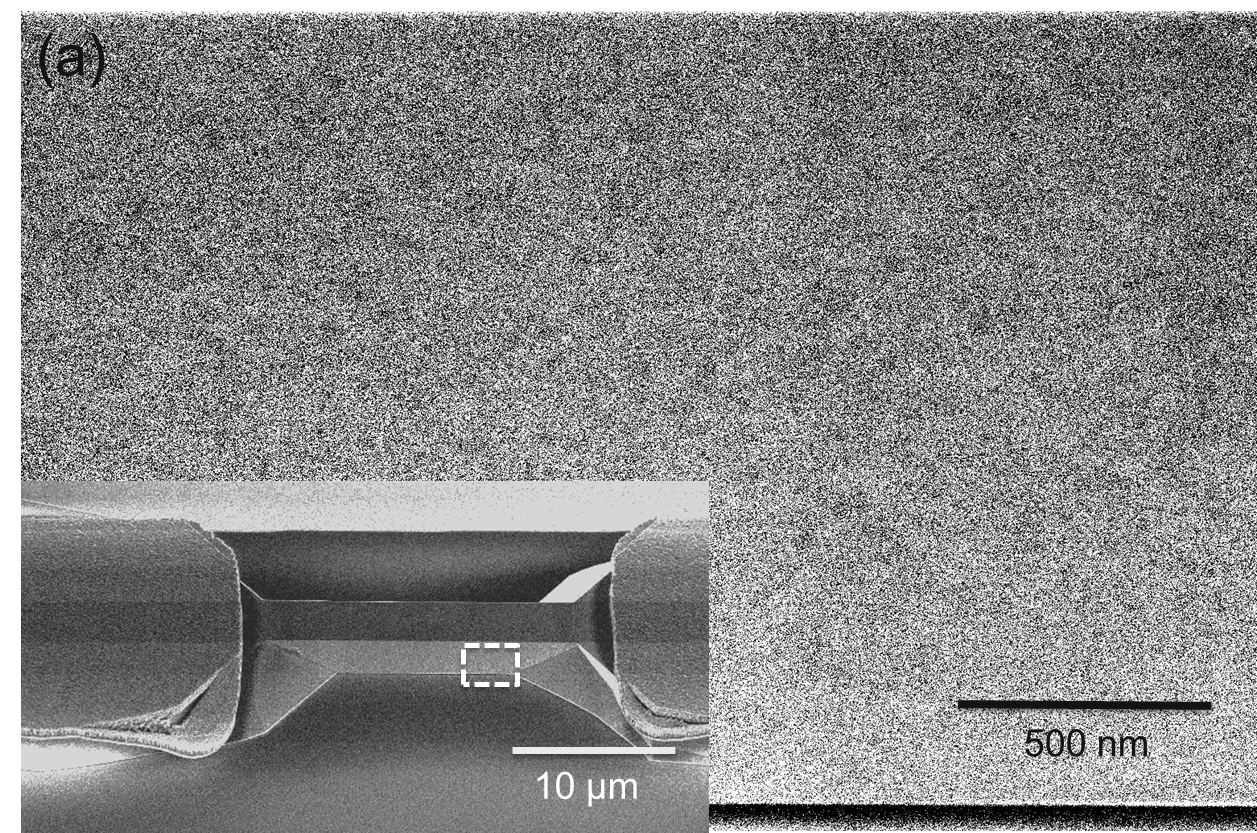

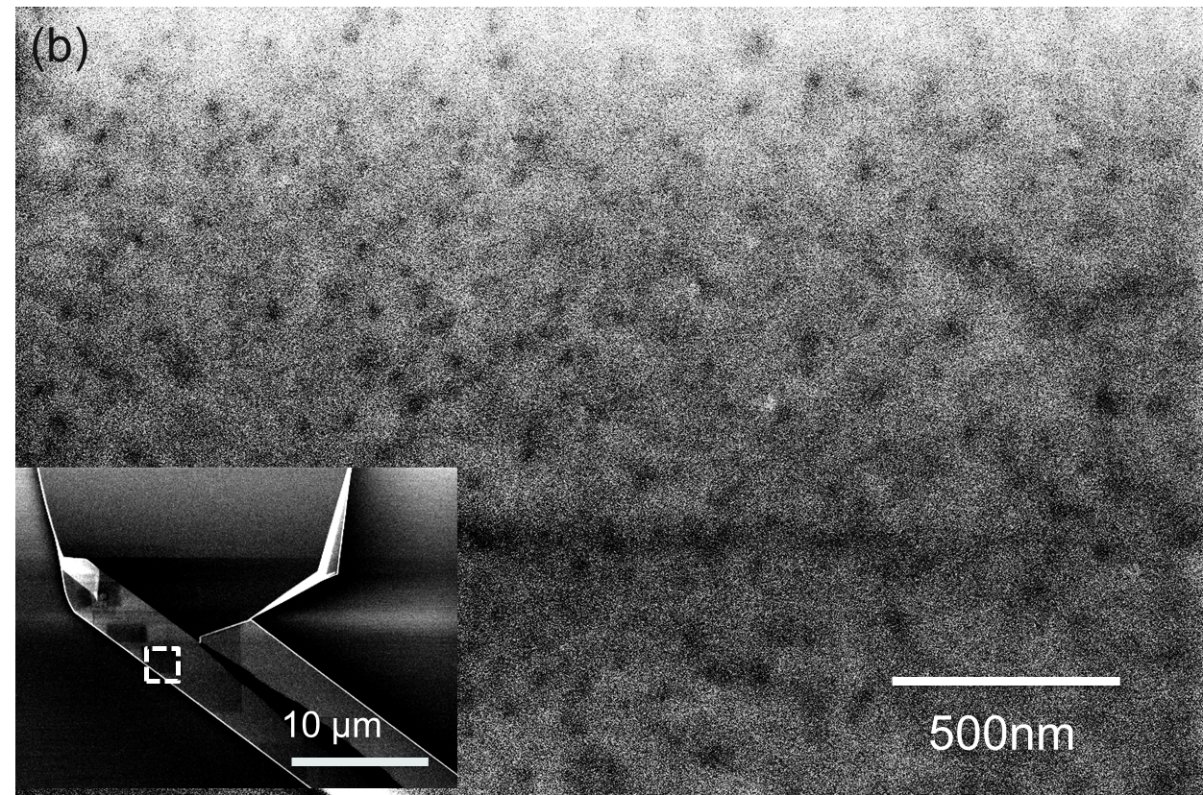


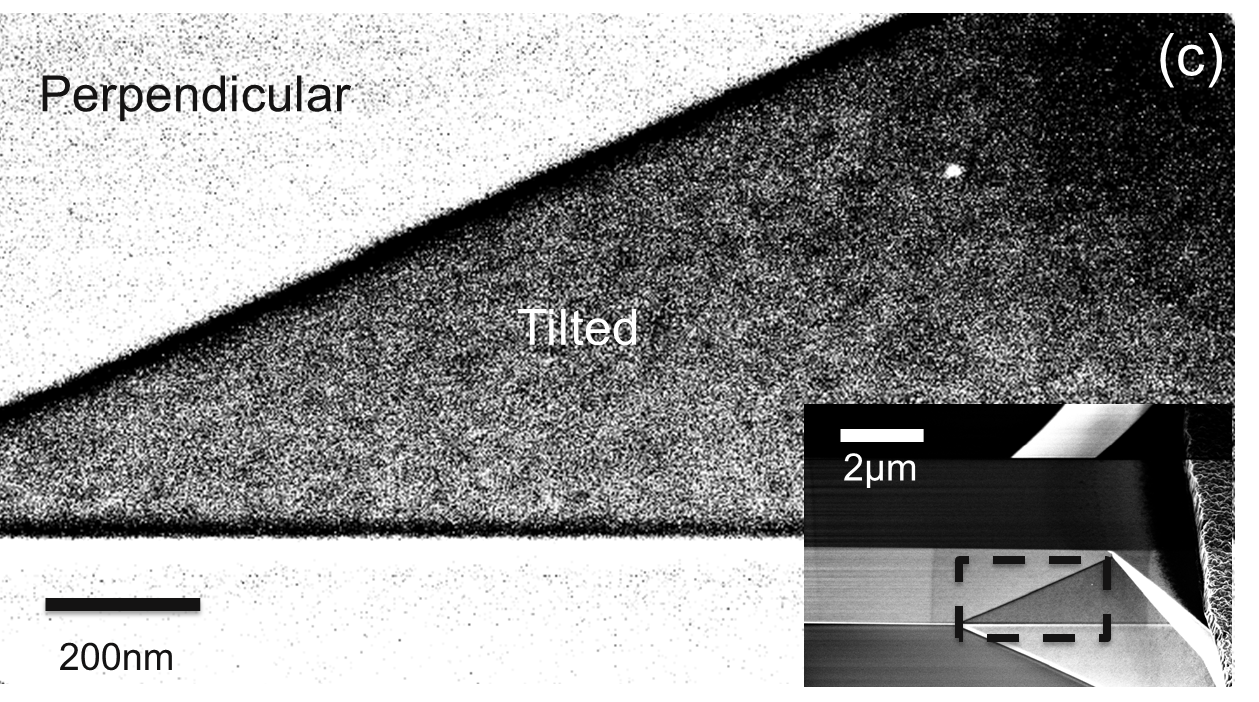


**Figure 2** (a) and (b) SEM pictures of the etched vertical and slanted walls, respectively. (c) SEM picture showing both of the etched walls simultaneously.

1. **Temperature dependent IV characteristics.**

In figs. 2 (a) and (b) representative temperature dependent IV curves for one rectangular and triangular resistors respectively are shown. These curves were used for the extrapolation of the resistance of the devices at each temperature through a linear fitting.


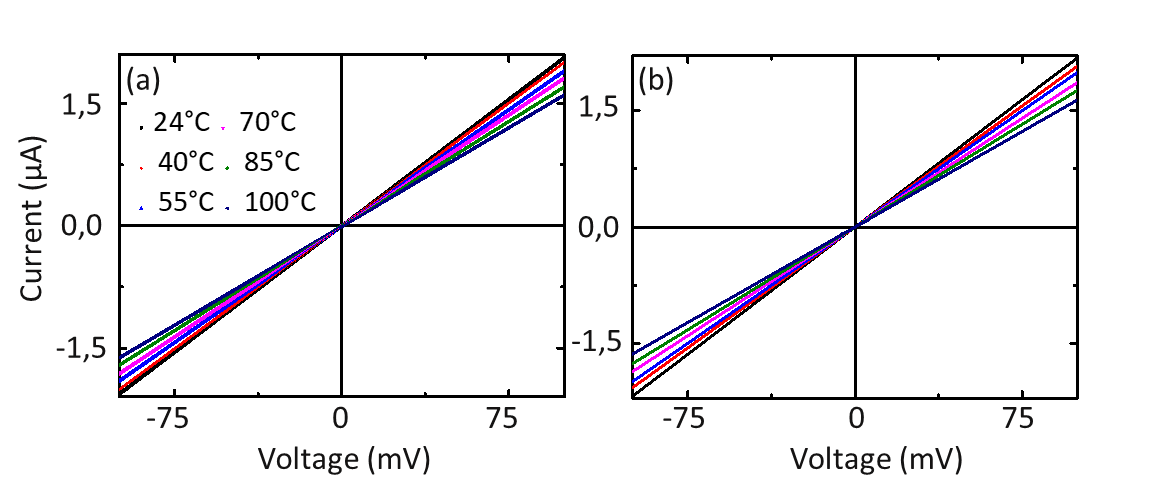


**Figure 3.** (a) and (b) Temperature dependent IV curves for a rectangular and triangular resistor respectively.

1. **Calculations of the accuracy**

To have information about the accuracy of our devices we considered the dependency of the temperature on the change of the resistance ($\Delta R$) and the first order thermal coefficient (*k*) as shown in eq. (1). When we determined these two parameters experimentally from linear fitting of the experimental data, they both were accompanied by an error. Taking this into account, we used the error propagation formula according to which the error on a quantity which depends on other quantities can be estimated from the square root of the sum of the fractional uncertainties on these latest ones. Therefore we applied eq. (2) to calculate the accuracy ($\delta T$). Here, $T_{RT}$ is the room temperature, and $\delta k$ and $\delta R$ are the errors on the first order temperature coefficient and on the resistance respectively.

1. $\Delta T= \frac{\Delta R}{k}$
2. $\delta T=T_{RT}\cdot\sqrt{\left( \frac{\delta R}{R} \right)^{2}+\left( \frac{\delta k}{k} \right)^{2}}$


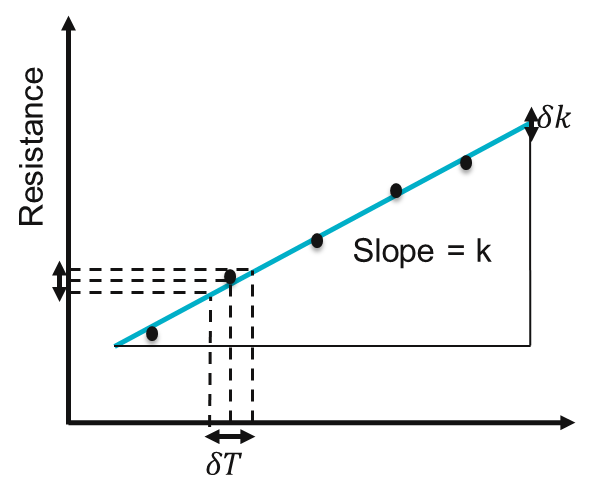
Figure 3 is a schematic of the significance of accuracy and its calculation.

**Figure 4.** Schematic of the quantities considered in the calculation of the accuracy.

1. **Comparison of the conductivity with literature data.**


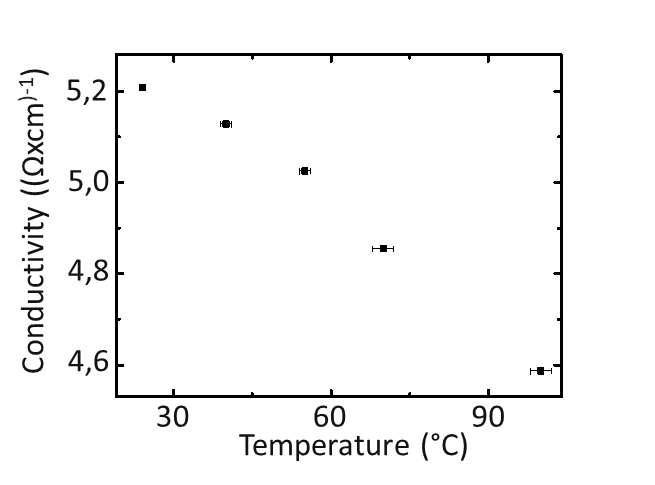
As stated in the paper the variation of the resistivity (therefore conductivity) of our devices with temperature was in agreement with literature data. We repeated the procedure explained in 3.1 to calculate the resistivity at each temperature and we calculated the conductivity as the inverse of the resistivity. Figure 4 shows the plot of the conductivity with temperature. These results were compared with literature^1^ and they were found to be in agreement within the 15%.

**Figure 5.** Plot of the calculated conductivity vs temperature.

**References**

^1^ Morin, F. J. and Maita, J. P. Electrical Properties of Silicon Containing Arsenic and Boron. *Physical Review* **96**, 28-35 (1954).
